# Transient Production of Human β-Glucocerebrosidase With Mannosidic-Type *N*-Glycan Structure in Glycoengineered *Nicotiana benthamiana* Plants

**DOI:** 10.3389/fpls.2021.683762

**Published:** 2021-06-07

**Authors:** Naphatsamon Uthailak, Hiroyuki Kajiura, Ryo Misaki, Kazuhito Fujiyama

**Affiliations:** ^1^International Center for Biotechnology, Osaka University, Osaka, Japan; ^2^Industrial Biotechnology Initiative Division, Institute for Open and Transdisciplinary Research Initiatives, Osaka University, Osaka, Japan; ^3^Cooperative Research Station in Southeast Asia, International Center for Biotechnology, Osaka University, Mahidol University, Bangkok, Thailand

**Keywords:** β-glucocerebrosidase, transient expression, *Nicotiana benthamiana*, *N*-glycosylation, plant-made pharmaceuticals, *N*-acetylglucosaminyltransferase I

## Abstract

Gaucher disease is an inherited lysosomal storage disorder caused by a deficiency of functional enzyme β-glucocerebrosidase (GCase). Recombinant GCase has been used in enzyme replacement therapy to treat Gaucher disease. Importantly, the terminal mannose *N*-glycan structure is essential for the uptake of recombinant GCase into macrophages via the mannose receptor. In this research, recombinant GCase was produced using *Agrobacterium*-mediated transient expression in both wild-type (WT) and *N*-acetylglucosaminyltransferase I (GnTI) downregulated *Nicotiana benthamiana* (ΔgntI) plants, the latter of which accumulates mannosidic-type *N*-glycan structures. The successfully produced functional GCase exhibited GCase enzyme activity. The enzyme activity was the same as that of the conventional mammalian-derived GCase. Notably, *N*-glycan analysis revealed that a mannosidic-type *N*-glycan structure lacking plant-specific *N*-glycans (β1,2-xylose and α1,3-fucose residues) was predominant in all glycosylation sites of purified GCase produced from ΔgntI plants. Our research provides a promising alternative plant line as a host for the production of recombinant GCase with a mannosidic-type *N*-glycan structure. This glycoengineered plant might be applicable to the production of other pharmaceutical proteins, especially mannose receptor targeted protein, for therapeutic uses.

## Introduction

Gaucher disease, one of the most common lysosomal storage disorders, is caused by the mutation of the *GBA1* gene, resulting in the defective activity of a lysosomal enzyme called glucocerebrosidase (GCase, β-glucosidase; EC: 3.2.1.45). Enzyme replacement therapy (ERT) is the most effective treatment for Gaucher disease type 1; it involves intravenous infusions of exogenous recombinant GCase to patients (Siebert et al., 2014; Stirnemann et al., 2017; Boer et al., 2020). Three drugs are commercially available: Imiglucerase (Cerezyme®, Genzyme Corporation), Velaglucerase alfa (VPRIV®, Shire Plc), and Taliglucerase alfa (Elelyso®, Pfizer). These drugs are recombinant GCases produced in Chinese hamster ovary (CHO) cells, human fibroblasts, and carrot suspension cells, respectively. Moreover, all are glycoproteins containing different *N*-glycan structures (Bennett and Fellner, 2018).

Glycosylation is the enzymatic transfer and attachment of glycans to specific sites on proteins. It is important for certain protein characteristics, including folding, solubility, stability, functionality, half-life, and quality control (Castilho and Steinkellner, 2012; Loos and Steinkellner, 2014; Schoberer and Strasser, 2018; Zhou and Qiu, 2019). Since recombinant GCase is taken up by macrophage via the mannose receptor, the terminal mannose *N*-glycan structure is essential for the translocation of commercial drugs into macrophages (Friedman et al., 1999). For this reason, the *N*-glycan structures of three commercially available GCases have been modified using different approaches in order to possess terminal mannose residues. First, Imiglucerase has been modified by exoglycosidase digestion during the post-production process, whereas mannosidic-type *N*-glycan of Velaglucerase alfa has been achieved by the addition of kifunensine, a mannosidase I inhibitor, into medium. On the other hand, Taliglucerase alfa has been modified by targeting GCase to the vacuole in order to produce the pauci-mannosidic structure (Van Patten et al., 2007; Tekoah et al., 2013; Kallemeijn et al., 2017). Although Imiglucerase is the leading brand, its manufacturing plants were shut down due to viral contamination in 2009, which resulted in a shortage of drugs for ERT (Hollak et al., 2010). Compared to those two drugs, Taliglucerase alfa is free from contamination by human pathogens and does not require post-production modification (Grabowski et al., 2014; Gupta and Pastores, 2017; Zimran et al., 2018). Even though drugs are commercially available, a large amount of recombinant GCase is required for ERT. Recently, administration of an ERT drug at a dosage of 60 Units/kg body weight every 2 weeks costs ~$128,000 to $544,000 per patient annually. Thus, Gaucher disease drugs have been reported to be among the most expensive drugs available (Joerg et al., 2007; Burnett and Burnett, 2020).

A plant-based expression platform has many advantages for the production of pharmaceutical recombinant proteins, including low cultivation costs, scale-up possibility, complex protein production ability with post-translational modifications, and a low risk of contamination by human pathogens (Paul and Ma, 2011; Moon et al., 2019; Burnett and Burnett, 2020; Shanmugaraj et al., 2020). After Taliglucerase alfa became the first plant-made pharmaceutical approved by the U.S. Food and Drug Administration in 2012, studies on the use of plants for pharmaceutical and non-pharmaceutical recombinant protein production have been increasing (Van Dussen et al., 2013; Tschofen et al., 2016). Many studies have focused on the production of recombinant GCase in plants including *Arabidopsis thaliana* (He et al., 2012), rice suspension culture (Nam et al., 2017; Jung et al., 2019), and *Nicotiana benthamiana* (Limkul et al., 2015, 2016; Naphatsamon et al., 2018). However, some studies did not report the *N*-glycan structure of recombinant GCase (Limkul et al., 2015; Nam et al., 2017), whereas one study revealed the majority of *N*-glycan structure containing residues of plant-specific glycan (β1,2-xylose and α1,3-fucose) (Naphatsamon et al., 2018). Those nonhuman glycosylations have the potential to induce immunogenic responses in human (Gomord et al., 2005, 2010; Montero-Morales and Steinkellner, 2018). *N*-glycan modification is one of the approaches to overcome this problem. Many plant-based glycoengineering studies have focused on the knockdown or knockout of the *N*-acetyltglucosaminyltransferase I (GnTI) gene, leading to mannosidic-type structures and to the absence of plant-specific glycan residues (Strasser, 2014; Limkul et al., 2016; Montero-Morales and Steinkellner, 2018).

*Agrobacterium*-mediated transient expression (agroinfiltration) is widely used to produce large amounts of protein due to its high expression level. It also has other advantages, including effectiveness, low-cost production, time saving and scalability (Moon et al., 2019). Notably, this system is useful for pandemic responses due to rapid gene introduction (Burnett and Burnett, 2020). For instance, the end product of an influenza vaccine was produced within 3 weeks by the Medicago company using plant transient expression (D'Aoust et al., 2010). Compared to other plants, *N. benthamiana* has been widely used for recombinant protein production because of its high biomass and susceptibility to a wide range of plant pathogens, the latter of which is useful for introducing foreign genes (Goodin et al., 2008).

In this study, we developed an alternative system to produce recombinant GCase with mannosidic-type *N*-glycan structure in WT and glycoengineered *N. benthamiana* plants (NbGNTI-RNAi7; ΔgntI) (Limkul et al., 2016) using *Agrobacterium*-mediated transient expression. After purification using two steps of column chromatography, purified recombinant GCase was analyzed by an enzymatic activity assay. The glycan structure of purified recombinant GCase was analyzed at three glycosylated positions. Our work demonstrated that recombinant GCase was successfully produced as an active glycoprotein with a mannosidic-type glycan structure.

## Materials and Methods

### Plants and Growth Conditions

Stable glycoengineered *gntI*-knockdown *N. benthamiana* plants [NbGNTI-RNAi7; ΔgntI) T7 were generated as described previously (Limkul et al., 2016). Seeds of ΔgntI plants were sterilized using PPM solution (Plant Preservative Mixture; Plant Cell Technology, USA) and selected on a Murashige and Skoog (MS) medium agar plate supplemented with hygromycin (30 mg/L). Germinated seedlings were transferred to soil pots and grown at 25°C under a 16 h light and 8 h dark condition for 4 weeks. Seeds of WT *N. benthamiana* were geminated on soil and grown under same condition as described above.

### Agrobacterium Strains and Plant Expression Vectors

**Figure 1:**
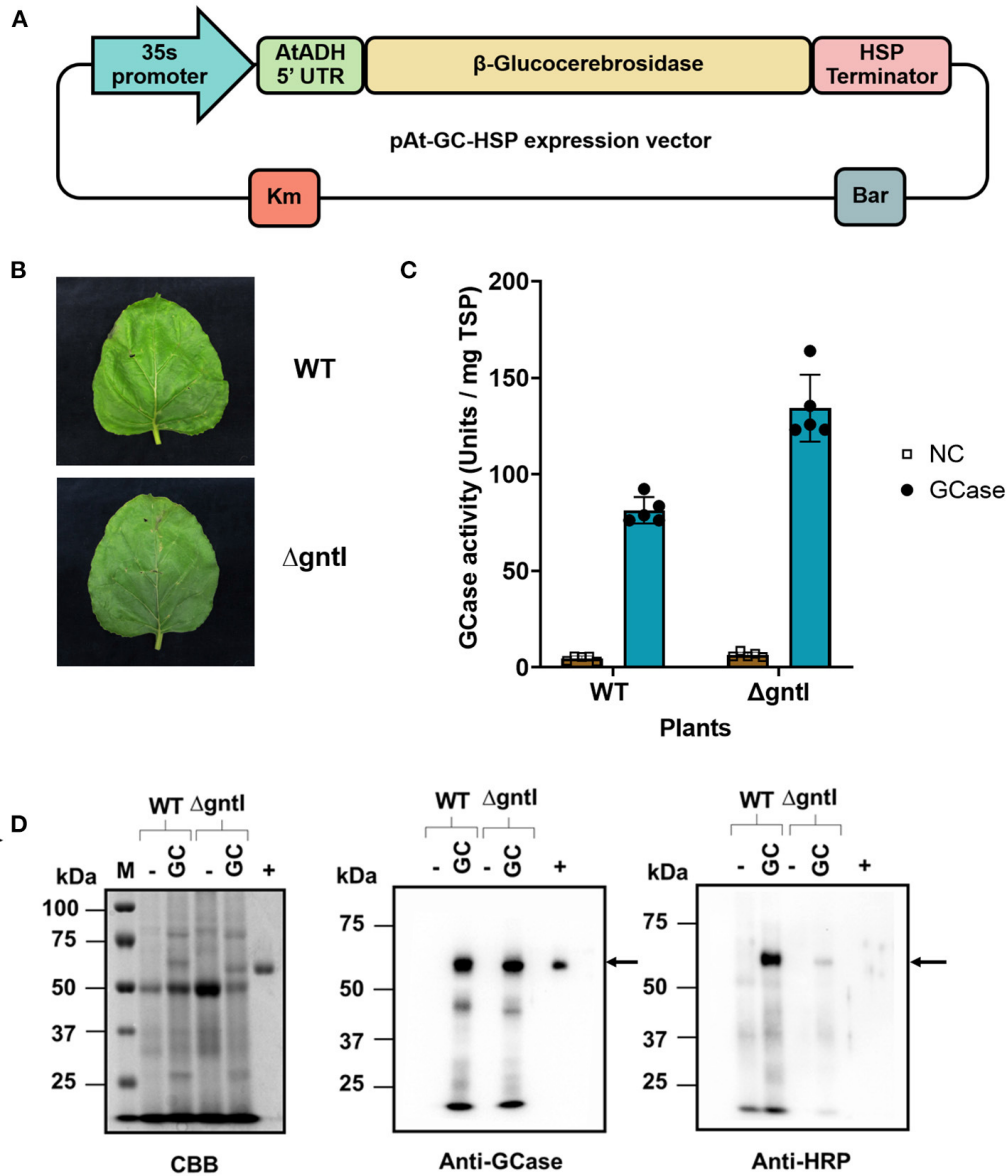
Transient production of recombinant GCase in wild-type (WT) and ΔgntI *N. benthamiana* plants. **(A)** The pAt-GC-HSP expression vector. 35S promoter: Cauliflower mosaic virus 35S RNA promoter; AtADH 5'UTR: 5'-UTR of *Arabidopsis* alcohol dehydrogenase (AtADH) gene; β-Glucocerebrosidase: β-glucocerebrosidase gene; HSP terminator: Transcription terminator from *A. thaliana* heat shock protein; Bar: bialaphos resistance gene; Km: Kanamycin resistance gene. **(B)** Infiltrated leaves and **(C)** GCase activity of crude protein from infiltrated WT and ΔgntI leaves at 7 days post-infiltration. NC: non-infiltrated leaves (negative control). The experiment was performed in five replications. Error bars represent the standard deviation (SD). **(D)** Total soluble protein: 10, 5, and 1 μg were loaded and analyzed by Coomassie Brilliant Blue (CBB) staining, anti-GCase blotting, and anti-HRP blotting, respectively. M, Precision Plus Protein standards marker; WT, wild-type *N. benthamiana*; ΔgntI, *gntI*-knockdown *N. benthamiana*; (-), non-infiltrated leaves (negative control); (+), Cerezyme®, a commercial GCase produced in Chinese Hamster Ovary (CHO) cells. Arrows represent the GCase band.

*Agrobacterium tumefaciens* strain LBA4404 harboring GCase expression plasmid (pAt-GC-HSP) was constructed previously (Limkul et al., 2015) (Figure 1A). A vector for the expression of RNA silencing suppressor 19 (p19) was kindly provided by Prof. Atsushi Takeda (Ritsumeikan University). The p19 vector was transformed into *A. tumefaciens* by electroporation. An *A. tumefaciens* transformant was selected using kanamycin (50 mg/L), rifampicin (50 mg/L), and streptomycin (50 mg/L).

### Agrobacterium-Mediated Transient Expression

A single colony of *Agrobacterium* was inoculated into 2xYT liquid medium supplemented with the appropriate antibiotics and cultivated at 28°C overnight. Two milliliters of culture was inoculated into 2xYT liquid medium (200 ml) supplemented with the same antibiotics and cultivated at 28°C overnight. Cells were collected by centrifugation at 4,000 × *g*. *Agrobacterium* containing different vectors (GCase or p19) was resuspended and mixed at a 1:1 ratio in infiltration buffer (10 mM MES, 10 mM MgSO_4_, 100 μM acetosyringone, pH 5.5) with OD_600_ of 0.5. The *Agrobacterium* mixture was incubated at room temperature for 2–4 h. Both WT and ΔgntI *N. benthamiana* plants were infiltrated using vacuum infiltration (Chen et al., 2013). Infiltrated plants were grown at 25°C under 16 h light and 8 h dark condition for 1 week.

### Optimization of Vacuum Infiltration

*Agrobacterium* culture and infiltration buffer were prepared as described above. For OD_600_ optimization, 4-week-old *N. benthamiana* plants were infiltrated in the *Agrobacterium* mixture with different OD_600_ values: 0, 0.25, 0.50, 0.75, and 1.0. Infiltrated plants were grown at 25°C under 16 h light and 8 h dark condition. Agroinfiltrated leaves were collected at 7 days post-infiltration (dpi). For the optimization of harvest time, *N. benthamiana* plants were infiltrated with the *Agrobacterium* mixture as described above (OD_600_ = 0.5). Agroinfiltrated leaves were collected at 5, 6, 7, and 8 days post-infiltration (dpi).

### Protein Extraction From Agroinfiltrated Leaves

The protein extraction method was modified from a previous study (Limkul et al., 2015). Briefly, leaves were homogenized by grinding with liquid nitrogen. Leaf powders were suspended in GCase extraction buffer (20 mM Tris-HCl pH 7.0, 150 mM NaCl, 0.5% taurocholic acid, and ethylenediaminetetraacetic acid (EDTA)-free Protease Inhibitor Cocktail (Roche Diagnostics GmbH, Germany) and centrifuged at 12,000 × *g* for 15 min. The supernatant was collected and analyzed by a GCase activity assay, a Bradford assay, SDS-PAGE, and Western blotting, as described below.

### GCase Activity Assay

The enzyme activity assay was performed as described previously (Limkul et al., 2015). Briefly, crude extract was co-incubated with GCase assay buffer (60 mM phosphate-citrate buffer pH 6.0, 4 mM β-mercaptoethanol, 1.3 mM EDTA, 0.15% Triton X-100, 0.125% taurocholic acid) and 0.2 mM of 4-methylumbelliferyl β-D-glucopyranoside (4-MUG; Wako, Japan) at 37°C for 1 h. The enzymatic reaction was terminated using cold glycine stop buffer (0.2 M glycine, 0.125 M sodium carbonate, pH 10.7). The fluorescence of the product, 4-methylumbelliferone (4-MU; Wako), was detected using an F-25000 fluorescence spectrophotometer (Hitachi High-Technologies, Japan) at λ_ex_ = 365 nm and λ_em_ = 460 nm. The amount of reaction product from the GCase activity assay was calculated using the 4-MU standard curve. An enzyme unit was defined as the amount of enzyme used to release 1 nmol of 4-MU/min. GCase enzyme activity was calculated by Units/mg of total soluble protein (TSP).

### SDS-PAGE and Western Blot Analysis

Crude extracted protein of agroinfiltrated leaves was separated by 10% sodium dodecyl sulfate-polyacrylamide gel electrophoresis (SDS-PAGE). Proteins were stained using Coomassie Brilliant Blue (CBB) following the manufacturer's instructions (Ready to Use; Nacalai Tesque, Japan). For Western blot analysis, proteins in polyacrylamide gel were transferred to a polyvinylidene difluoride (PVDF) membrane (Millipore, USA). The membrane was incubated with skim milk (5%) in PBS-T (1.47 mM KH_2_PO_4_, 10 mM Na_2_HPO_4_, 2.7 mM KCl, 137 mM NaCl, 0.05% Tween-20, pH 7.4) at room temperature for 1 h. Then it was further incubated with 1:5,000 diluted polyclonal anti-glucocerebrosidase (GCase) antibody from rabbit (Sigma-Aldrich, USA) or polyclonal anti-horseradish peroxidase from rabbit (Sigma-Aldrich) with 1:12,500 dilution at 25°C for 1 h. The membrane was washed with PBS-T and further incubated with the 1:5,000 diluted anti-rabbit IgG, HRP-linked whole antibody (GE Healthcare, Japan) at 25°C for 1 h. After washing with PBS-T, proteins were detected by incubation with Luminata^TM^ (Millipore) at room temperature for 5 min using the iBright Imaging System (Invitrogen, USA).

### GCase Purification From Agroinfiltrated Leaves

Purification was performed as described previously (Limkul et al., 2016). Agroinfiltrated leaves were homogenized by grinding with liquid nitrogen. Leaf powders were suspended in GCase extraction buffer and incubated on ice for 20 min. Crude extracts were collected using centrifugation (10,000 x *g* for 20 min) and loaded into a Concanavalin (Con A)- Agarose column (Vector Laboratories, USA) using a peristaltic pump (Perista Pump SJ-1211; ATTO, Japan) with a recycling system at 4°C for 12 h. The column was washed with Con A buffer (500 mM NaCl, 1 mM MgCl_2_, 1 mM MnCl_2_, 1 mM CaCl_2_ in 20 mM Tris-HCl pH 7.0). Proteins were eluted with GCase assay buffer containing 300 mM α-methyl-D-mannoside (Nacalai Tesque). Each eluted fraction was analyzed by a GCase enzymatic assay. All eluted fractions with GCase activity were collected and resuspended in 2 M NaCl. Protein solution was loaded onto a Phenyl 650M column (Tosoh, Japan). Proteins were eluted by decreasing the NaCl concentration. Each elution fraction was analyzed using the GCase enzyme assay. Active fractions were dialyzed and concentrated using an Amicon® Ultra centrifugal filter (Millipore). Concentrated protein was analyzed by the GCase activity assay, Bradford assay, SDS-PAGE, and Western blotting.

### *N*-Glycan Analysis of Purified GCase

Purified GCase from agroinfiltrated leaves was used for *N*-glycan analysis as described previously (Limkul et al., 2016). The purified GCase was separated using SDS-PAGE and stained by PageBlue protein stain solution (Thermo Fisher Scientific, USA). In-gel digestion was performed as follows: GCase bands were de-stained using 1:1 v/v of acetonitrile and 50 mM NH_4_HCO_3_ at room temperature overnight. The proteins were then digested with Trypsin Gold (Promega, USA) in ProteaseMAX^TM^ surfactant solution (Promega) at 50°C for 1 h. The reaction was terminated by 1% trifluoroacetic acid in 60% acetonitrile. Peptides were extracted from gel and evaporated at 30°C for 3 h. Peptides were dissolved in 0.1% formic acid and applied to an ESI-Qq-TOF mass spectrometer (microTOF-Q II, Bruker Daltonics, Germany). The conditions used for *N*-glycan analysis were as described previously (Limkul et al., 2016). Briefly, a nano-LC system (120 series; Agilent Technologies) was used with a trap column (5 μ, 0.3 × 5 mm) and an analytical column (3.5 μ, 0.075 x 150 mm). Both 0.1% formic acid in water and 0.1 % formic acid in acetonitrile were used as the mobile phase. Peptides were trapped in the trapped column and eluted at a flow rate of 0.6 μL/min. The system was completely controlled by microTOF control software (Bruker Daltonics). *N*-Glycan structures were analyzed using DataAnalysis version 4.0 (Bruker Daltonics) with both MS and MS/MS modes.

## Results

### Transient Production of Recombinant GCase in WT and ΔgntI *N. benthamiana* Plants

To produce recombinant GCase in *N. benthamiana* plants, 4-week-old plants were infiltrated with *Agrobacterium* mixture harboring GCase and p19 vectors at an OD_600_ of 0.5. At 7 days post-infiltration, leaf samples were collected for protein extraction and crude proteins were further analyzed using an enzymatic assay and protein blotting.

After infiltration, no necrosis was observed from the infiltrated WT or ΔgntI leaves (Figure 1B). The enzymatic assay detected GCase activity from crude protein of both plants (Figure 1C). The GCase produced in ΔgntI plants showed higher GCase activity (134.3 ± 17.3 U/mg TSP) than that produced in WT plants (81.5 ± 6.9 U/mg TSP). On the other hand, non-infiltrated WT and ΔgntI leaves (negative control) showed low GCase activity (4.7 ± 1.3 and 6.5 ± 1.4 U/mg TSP in WT and ΔgntI plants, respectively). The GCase activity detected in the negative control was considered as an effect of plant-produced β-glucosidase (Seshadri et al., 2009; Pankoke et al., 2013). Crude proteins were further analyzed by CBB staining and Western blotting compared to Cerezyme®, a commercial GCase produced in CHO cells as a positive control (Figure 1D). A GCase band was detected using anti-GCase antibody from crude protein extracted from agroinfiltrated WT and ΔgntI leaves with a molecular weight of ~58 kDa. On the other hand, no GCase band was detected from the negative control. Additionally, plant-specific glycans were determined using anti-HRP antibody blotting. Anti-HRP antibody binds to plant specific *N*-glycan residues, including β1,2-xylose and α1,3-fucose residues (Jin et al., 2008; Iskratsch et al., 2009; Paschinger et al., 2009). In this work, higher-intensity bands were detected from crude protein produced in WT plants than that of ΔgntI plants using anti-HRP antibody. This implies that the GCase produced from ΔgntI plants contains less plant specific glycan residues compared to GCase produced from WT plants. These results indicated that the functional recombinant GCase was successfully produced in both WT and ΔgntI plants. Furthermore, ΔgntI-infiltrated leaves had fewer plant-specific *N*-glycan structures than WT leaves.

### Optimization of Infiltration Condition for Transient GCase Production

To obtain a large amount of recombinant GCase, infiltration conditions, including the optical density (OD_600_) of *Agrobacterium* mixture and the harvest time, were optimized in this study. WT and ΔgntI plants were infiltrated using the *Agrobacterium* mixture with different OD_600_ values: 0.25, 0.50, 0.75, and 1.0. Leaf samples were collected at 7 days post-infiltration.

**Figure 2:**
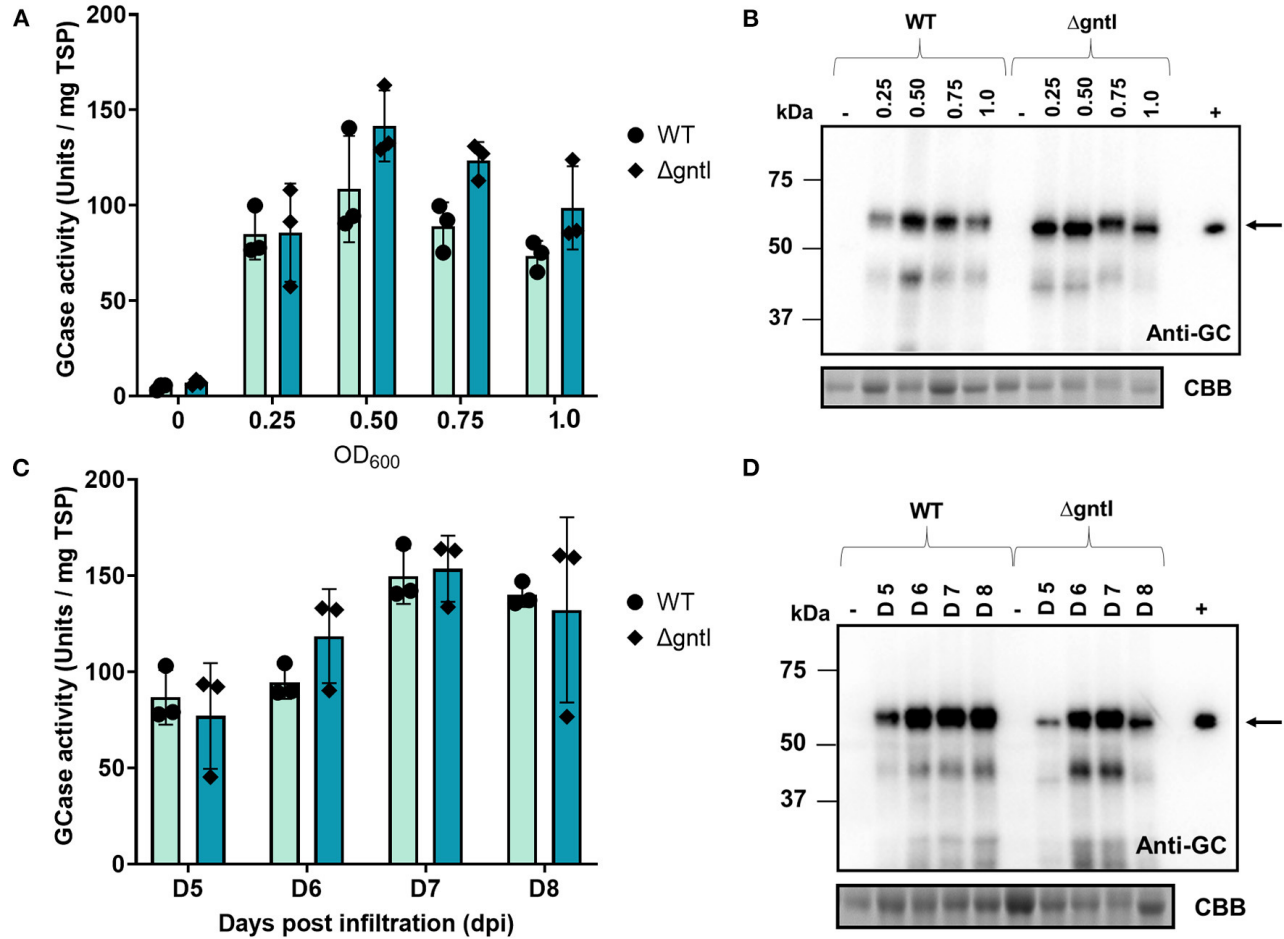
Optimization of GCase transient production. GCase activity of crude protein from wild-type (WT) and ΔgntI leaves infiltrated with **(A)** different OD_600_ values and **(C)** different days post-infiltration. Total soluble proteins: 5 and 10 μg were loaded and analyzed using anti-GCase antibody and CBB staining, respectively **(B,D)**. Error bars represent the standard deviation (SD). The experiment was performed in three replications. (-), negative control from non-infiltrated leaves; (+), Cerezyme®; WT, wild-type *N. benthamiana*; ΔgntI, glycoengineered *N. benthamiana*.

Crude extracted at an OD_600_ of 0.50 showed the highest GCase activity in both WT and ΔgntI plants (108.5 ± 27.9 and 139.5 ± 19.7 U/mg TSP, respectively), followed by that extracted at an OD_600_ of 0.75 (Figure 2A). However, the difference was not significant. Regarding the Western blotting result, OD_600_ values of 0.50 and 0.75 revealed higher intensities of GCase bands compared to OD_600_ values of 0.25 and 1.0 (Figure 2B). Because it had the highest GCase activity, OD_600_ of 0.5 was chosen as the optical density for GCase production. At harvest time, infiltrated leaf samples were collected at 5, 6, 7, and 8 days post-infiltration (dpi). The highest GCase activity was observed at 7 dpi in both WT (149.7 ± 14.5 U/mg TSP) and ΔgntI (153.6 ± 17.2 U/mg TSP) plants (Figure 2C). Additionally, GCase bands detected in anti-GCase blotting at 5 and 8 dpi showed lower intensities compared to those at 6 and 7 dpi (Figure 2D). Therefore, 7 dpi was chosen as the collection day for GCase purification.

### GCase Purification From Infiltrated WT and ΔgntI Leaves

Two types of column chromatography were used for GCase purification: Concanavalin (Con A) affinity chromatography and phenyl hydrophobic (P650M) chromatography. The binding between Con A and mannose residues of *N*-glycan structure have been reported (Maupin et al., 2012). After purification, GCases from infiltrated WT and ΔgntI leaves were analyzed using the enzymatic assay and protein blotting.

**Figure 3:**
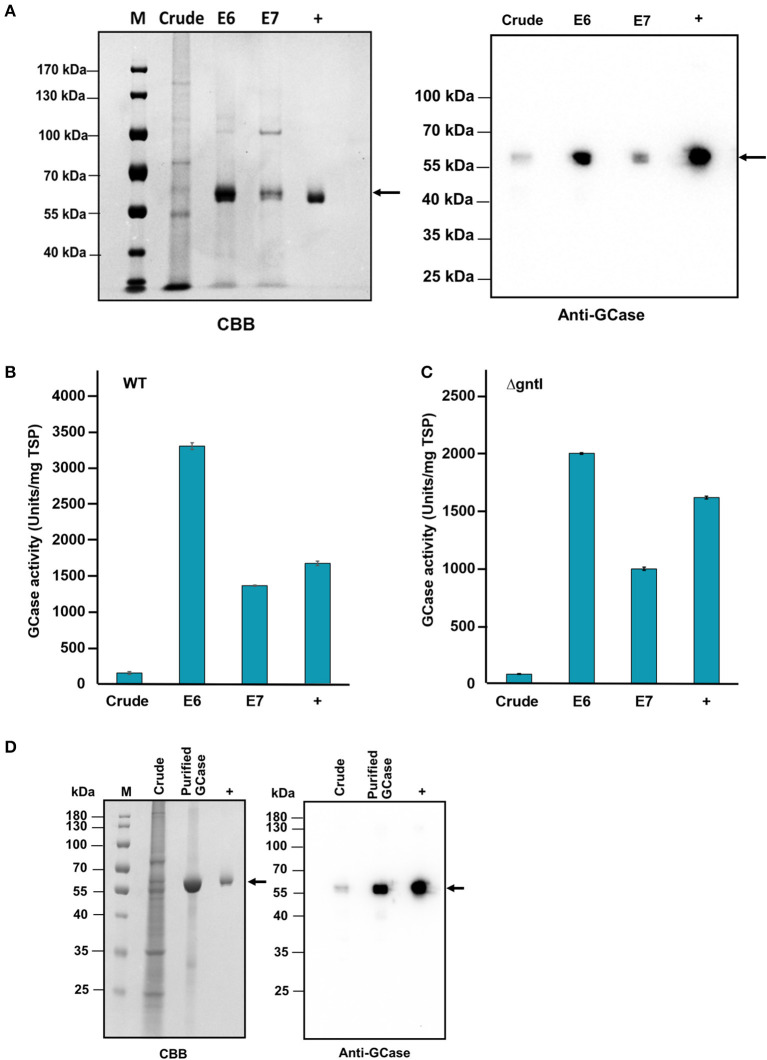
GCase purification from infiltrated wild-type (WT) and ΔgntI *N. benthamiana* leaves. **(A)** Total proteins from infiltrated WT leaves (E6 and E7); 2.5 μg and 100 ng were loaded and analyzed by CBB staining and Western blotting, respectively. **(B)** GCase activity of crude protein and purified GCase from infiltrated WT leaves: elution fractions 6 and 7. Error bars represent the standard deviation (SD) (*N* = 3). **(C)** GCase activity levels of crude protein and purified GCase from infiltrated ΔgntI leaves: elution fractions 6 and 7 (E6 and E7). Error bars represent the standard deviation (SD) (*N* = 3). **(D)** CBB staining and Western blot analysis of crude protein and purified GCase from infiltrated ΔgntI leaves elution fraction 6. M, PageRuler™ protein ladder; Crude, crude protein; Purified GCase, purified GCase from Phenyl 650M elution fractions 6 (E6); +, Cerezyme®.

With the infiltrated WT leaves, purified GCase bands were detected from P650M elution fractions 6 and 7 on CBB staining and Western blotting (Figure 3A). The GCase band was ~58 kDa, which corresponded to the size of the positive control, Cerezyme®. Additionally, no band was detected from the negative control, crude protein extracted from non-infiltrated leaves. The specific enzyme activity was measured using GCase activity assay. Purified GCase hydrolyzed the substrate, 4-MUG to glucose and 4-MU which was detected using a fluorescence spectrophotometer. The 4-MU standard curve was used to calculate the amount of purified GCase. The GCase-specific activities of purified GCase from eluted fractions 6 and 7 were ~3301.1 ± 48.4 and 1360.7 ± 8.9 U/mg TSP, respectively (Figure 3B). As expected, the GCase-specific activities of both eluted fractions were higher than that of crude protein (149.4 ± 18.3 U/mg TSP). The amounts of purified GCase from infiltrated WT leaves were ~5.4 μg and 2.7 μg in eluted fractions 6 and 7, respectively. The productivity of purified GCase was about 0.45 μg/g of fresh leaves.

On the other hand, purified GCase from infiltrated ΔgntI leaves was detected from P650M eluted fractions 6 and 7. The specific activities of purified GCase from eluted fractions 6 and 7 were 2001.7 ± 7.7 and 1,000.0 ± 13.8 U/mg TSP, respectively (Figure 3C). These numbers are much higher than that from the crude protein (84.7 ± 3.2 U/mg TSP). Additionally, a purified GCase band from eluted fraction 6 was detected on CBB staining and Western blotting compared to the positive control (Figure 3D). Different amounts of purified GCase from infiltrated ΔgntI leaves were determined from eluted fractions 6 and 7 (9.8 and 17.4 μg, respectively) with an approximate productivity of 2.6 μg/g of fresh leaves.

### *N*-Glycan Analysis of Purified GCase From Infiltrated Leaves

To analyze the *N*-glycan structures of purified GCase from both WT and ΔgntI samples, trypsin-digested glycopeptides were analyzed using nanoLC-MS/MS.

**Figure 4:**
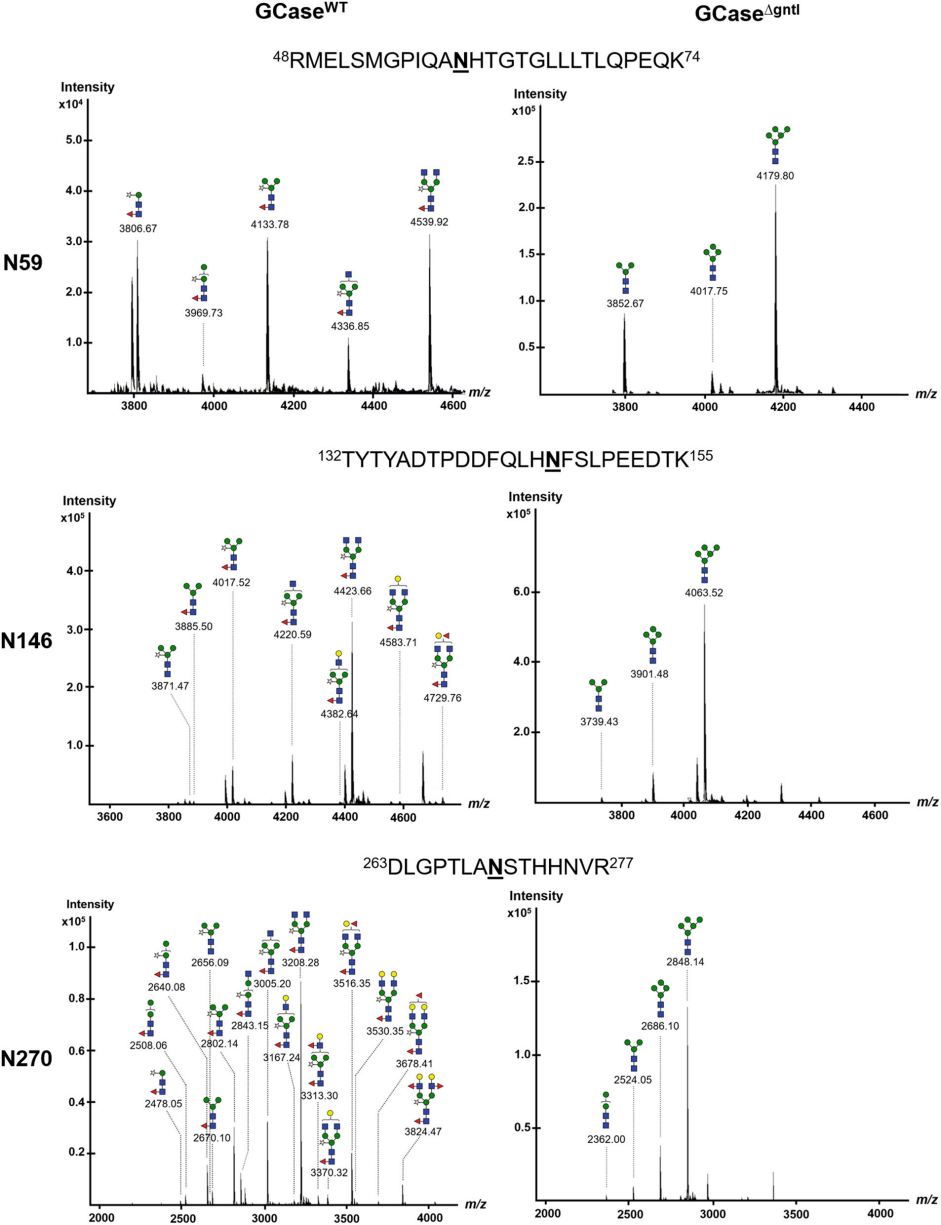
Nano LC–MS spectra of glycopeptides derived from purified GCase produced in wild-type (WT) and ΔgntI-infiltrated leaves. Glycan structures of glycopeptides are shown at different *N*-glycosylation sites (N59, N146, and N270).

Three *N*-glycosylation sites (N270, N146, and N59) were detected in purified GCase from both WT and ΔgntI plants (Figure 4). All possible *N*-glycan structures of each position were analyzed from deconvoluted MS spectra (Supplementary Figures 1–4). The composition percentage at each *N*-glycosylation site was also summarized (Table 1). In the case of WT GCase, GN2M3XF was the predominant structure at positions 59, 146 and 270 with percentages of 29.7%, 63.0%, and 38.2%, respectively. Additionally, GCase at positions N270 (100%), N146 (100%), and N59 (100%) was a *N*-glycosylated with at least one plant-specific *N*-glycans (β1,2-xylose and/or α1,3-fucose residues). On the other hand, Man5 was detected as the predominant structure at the N59 (89.2%), N146 (85%), and N270 (73.0%) positions in ΔgntI GCase. Additionally, the mannosidic-type structure without plant-specific glycan predominated in all positions, including N59 (100%), N146 (100%), and N270 (100%). These results demonstrated that recombinant GCase was successfully produced in ΔgntI plants with mannosidic-type *N*-glycan structures.

**Table 1:** Composition of *N*-glycan structures on purified GCases from wild type and ΔgntI agroinfiltrated plants at three *N*-glycosylation sites (N59, N146, and N270).

**Abbreviation**	**Structure**	**Amount of composition (%)**					
		**N59**		**N146**		**N270**	
		**WT**	**ΔgntI**	**WT**	**ΔgntI**	**WT**	**ΔgntI**
MXF	ManXylFuc	28.0	0	0	0	0.7	0
M2F	Man2Fuc	0	0	0	0	1.6	0
M2XF	Man2XylFuc	3.6	0	0	0	5.9	0
M3X	Man3Xyl	0	0	1.3	0	0.7	0
M3F	Man3Fuc	0	0	1.0	0	2.4	0
M3XF	Man3XylFuc	28.5	0	13.3	0	13.1	0
GNM2XF	GlcNAcMan2XylFuc	0	0	0	0	5.3	0
GNM3XF	GlcNAcMan3XylFuc	10.2	0	17.3	0	14.0	0
GAGNM3XF	GalGlcNAcMan3XylFuc	0	0	0.8	0	0.9	0
GN2M3XF	GlcNAc2Man3XylFuc	29.7	0	63.0	0	38.2	0
GAFGNM3XF	GalFucGlcNAcMan3XylFuc	0	0	0	0	1.6	0
GAGN2M3XF	GalGlcNAc2Man3XylFuc	0	0	0.9	0	1.8	0
GAFGN2M3XF	GalFucGlcNAc2Man3XylFuc	0	0	2.4	0	8.7	0
GA2GN2M3XF	Gal2GlcNAc2Man3XylFuc	0	0	0	0	1.1	0
GA2FGN2M3XF	Gal2FucGlcNAc2Man3XylFuc	0	0	0	0	0.6	0
GA2F2GN2M3XF	Gal2Fuc2GlcNAc2Man3XylFuc	0	0	0	0	3.4	0
**Plant-type structure (%)**		**100**	**0**	**100**	**0**	**100**	**0**
M2	Man2	0	0	0	0	0	1.9
M3	Man3	0	1.0	0	2.0	0	4.9
M4	Man4	0	9.8	0	13.0	0	20.2
M5	Man5	0	89.2	0	85.0	0	73.0
**Mannosidic-type structure (%)**		**0**	**100**	**0**	**100**	**0**	**100**

## Discussion

A large amount of recombinant GCase is required for the ERT treatment of Gaucher disease type 1 (Burnett and Burnett, 2020). Since exogenous GCase is translocated into macrophages via the mannose receptor, the mannose terminal *N*-glycan structure is essential for recombinant GCase (Friedman et al., 1999). In this work, we investigated the impact of *gntI*-downregulated *N. benthamiana* plants (ΔgntI) as a host for the transient expression of recombinant GCase with a mannosidic-type *N*-glycan structure.

Our results demonstrated that recombinant GCase was successfully produced in WT and ΔgntI *N. benthamiana* plants (Figure 1). Since *Agrobacterium*-infiltration conditions usually affect the transformation efficiency, resulting in different expression levels of recombinant protein (Hanittinan et al., 2020), two main factors, *Agrobacterium* cell density and harvest time, were optimized to produce a large amount of GCase with biological activity. Previous studies demonstrated that too low cell density might lead to insufficient *Agrobacterium* infection, whereas too high density may cause necrosis (Kagale et al., 2012; Leuzinger et al., 2013; Yan et al., 2019). Additionally, the harvesting time also affects the necrosis and amount of protein production (Kim et al., 2021). Our work revealed that the *Agrobacterium* cell density (OD_600_) of 0.5 and the harvest time of 7 dpi were the optimal conditions for GCase transient production, as they resulted in the highest GCase enzyme activity in both WT and ΔgntI-infiltrated leaves (Figure 2). Notably, the crude GCase activity in our work was higher than in previous studies: 65.5 U/mg TSP (Limkul et al., 2016), 24 U/mg TSP (He et al., 2012), 44.5 U/mg TSP (Limkul et al., 2015), and 81.4 U/mg TSP (Naphatsamon et al., 2018). On the other hand, the low GCase activity observed in the negative control was attributed to internal β-glucosidases produced in plants, which plays important roles in plant defense systems. This enzyme is also related to plant physiology, including cell wall degradation and to the activation of both phytohormones and chemical compounds (Morant et al., 2008; Pankoke et al., 2013). Internal β-glucosidases were also reported previously (Naphatsamon et al., 2018). Our results indicated that this system has the potential to be competitive for recombinant GCase production with high levels of biological activity.

Two different GCase purification strategies have been reported: hexa histidine-tag (His-tag) and a three-step purification method (Nam et al., 2017; Jung et al., 2019). However, the removal of his-tag and the multiple steps required for purification may increase the production cost. In this study, recombinant GCase was successfully purified as a single band using two types of column chromatography (Con A and P650M columns) adapted from our current works (Limkul et al., 2015, 2016; Naphatsamon et al., 2018) (Figure 3). The specific activity levels of purified GCase from infiltrated WT and ΔgntI leaves are competitive with those of the recombinant GCase produced in WT transgenic *N. benthamiana* plants (2641 U/mg TSP) and *N. benthamiana* root culture (1618 U/mg TSP) from previous studies (Limkul et al., 2015; Naphatsamon et al., 2018). Although recombinant GCase produced in stable glycoengineered *N. benthamiana* revealed higher specific activity (3063 U/ mg TSP) (Limkul et al., 2016), our recombinant GCase had a higher composition of the mannose terminal *N*-glycan structure, which we discuss below.

Three commercially available recombinant GCases have been approved for the ERT treatment of Gaucher disease type I. The drugs are administered to patients by IV infusions and taken up into macrophages via the mannose receptor (Stirnemann et al., 2017). Moreover, GCase is translocated into lysosome using a lysosomal membrane protein called LIMP-2 (Zhao et al., 2014). It has been demonstrated that the mannose terminal *N*-glycan structure is essential for the macrophage uptake of ERT drugs (Van Patten et al., 2007). Therefore, the present study aimed to produce recombinant GCase with a mannosidic terminal structure and few plant-specific *N*-glycans. Our method relies on the knockdown of the *gntI* gene encoding the key enzyme for the mannose-5 terminal *N*-glycan structure (Strasser, 2014; Montero-Morales and Steinkellner, 2018; Amann et al., 2019). Analysis of the *N*-glycan composition of purified GCase from ΔgntI plants revealed that the mannose-5 terminal (Man5) structure predominated in all glycosylation sites: N59, N146, and N270 (Figure 4 and Table 1). Notably, these percentages of mannosidic-type *N*-glycan structure without plant-specific glycans were higher than those of the purified GCase produced from stable glycoengineered *N. benthamiana* (ranging from 73.3 to 85.5%) and *A. thaliana* complex-glycan-deficient (*cgl*) seeds (85%) in previous studies (He et al., 2012; Limkul et al., 2016). Additionally, the diverse structure and different degree of *N*-glycosylation at N59, N146, and N270 sites were considered to be a microheterogeneity caused by the inefficient enzymatic pathways of the glycoprotein biosynthesis (Zacchi and Schulz, 2016). These results indicated that our ΔgntI *N. benthamiana* plants have the potential to produce recombinant GCase with a mannosidic-type *N*-glycan structure without plant-specific *N*-glycans.

In our previous study, recombinant GCase was produced in the stable transgenic *N. benthamiana* plant (Limkul et al., 2016). Compared to the stable expression, our transient expression system provided ~2.3-fold higher crude GCase activity. The increase of crude GCase activity resulted from *Agrobacterium*-mediated transient expression which is more efficient than that of stable expression by the gene integration (Burnett and Burnett, 2020). Additionally, the *N*-glycan structures of purified GCase produced from both stable and transient expression systems were different.

In the case of WT plants, the predominant *N*-glycan structure of purified GCase from stable and transient plants were M3XF and GN2M3XF, respectively. The high amount of GN2M3XF structure may be caused by the rapid production in transient system. In general, two terminal GlcNAc residues of the GN2M3XF structure are cleaved by plant β-hexosaminidases (HEXOs), resulting in the M3XF structure (Altmann et al., 1995; Bosch et al., 2013). In this work, the recombinant GCase was transiently produced in a short time. Therefore, cellular stress may have been induced and HEXOs activity may not have been sufficient to completely convert the GN2M3XF into the M3XF structure. Additionally, the GA2F2GN2M3XF structure was found only in purified GCase produced in transient system, corresponding with previous report on the structures of secreted glycoprotein mostly found in the apoplast (Bosch et al., 2013).

In the case of ΔgntI plants, GCase produced through transient expression had mannosidic structures only (100%). On the other hand, some plant-type structures were remained in the purified GCase from the stable expression (ranging from 14.5 to 26.7%). Therefore, the transient expression in ΔgntI plants has a higher potential to produce recombinant GCase with mannosidic *N*-glycan structures.

Furthermore, this plant can be applied as a host for the production of other pharmaceutical proteins, especially mannose-targeted proteins, for therapeutic uses. The interaction between glycosylated proteins and carbohydrate-binding proteins (lectins) is important for cellular recognition processes, including cellular migration, enzyme trafficking, and immunogenic function (Irache et al., 2008). Several mannosylated structures have been considered as promising devices for drug delivery or vaccination due to their ability to bind the mannose receptor, which is highly expressed on the surfaces of immune cells such as macrophages and dendritic cells (Keler et al., 2004).

## Conclusion

Our study demonstrated that the ΔgntI *N. benthamiana* plant can be used for the transient production of recombinant GCase with a mannosidic-type *N*-glycan structure. Furthermore, this glycoengineered plant-based transient expression system has potential as an alternative platform for the production of other recombinant therapeutic proteins. This plant is also potentially useful for therapeutic uses during pandemics and outbreaks by virtue of to their rapid response.

## Data Availability Statement

The raw data supporting the conclusions of this article will be made available by the authors, without undue reservation.

## Author Contributions

KF supervised and conceived the original idea of this work. NU designed and performed the experiments with technical support from HK and wrote the manuscript with support from KF, HK, and RM. All authors approved the final manuscript.

## Conflict of Interest

The authors declare that the research was conducted in the absence of any commercial or financial relationships that could be construed as a potential conflict of interest.
