# Supplementary material for: Transient Production of Human β-Glucocerebrosidase With Mannosidic-Type N-Glycan Structure in Glycoengineered Nicotiana benthamiana Plants
Source: Front Plant Sci. 2021 Jun 7;12:683762. doi: 10.3389/fpls.2021.683762 (PMC8215604; doi:10.3389/fpls.2021.683762)
Supplement: Supplementary file 1 [file Data_Sheet_1.PDF]

Supplementary Material

Supplementary Figures

Abbreviation	Structure	Mass-to-charge ratio (*m/z*)					
		N59		N146		N270	
		Theoretical	Observed	Theoretical	Observed	Theoretical	Observed
Core+GN	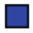	3166.6	3166.5	3050.3	3050.2	1834.9	1834.8
GN2	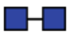	3369.6	3369.6	3253.3	3253.3	2037.9	2037.9
MGN2	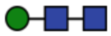	3531.6	3530.6	3415.3	3415.4	2199.9	2199.9
M2GN2	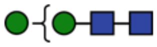	3693.6	3693.7	3577.3	3557.4	2361.9	2362.0
M3GN2	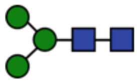	3855.6	3854.7	3739.3	3739.4	2523.9	2524.0
M3FGN2	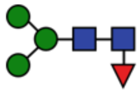	4002.6	4002.7	3886.3	3885.5	2670.9	2670.1
M3FXGN2	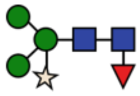	4134.6	4133.8	4018.3	4017.5	2802.9	2802.1

Supplementary Figure 1. Quantification of *N*-glycan structures on the purified GCase produced from wild type agroinfiltrated *N. benthamiana* leaves (MS/MS mode)

Abbreviation	Structure	Mass-to-charge ratio (*m/z*)					
		N59		N146		N270	
		Theoretical	Observed	Theoretical	Observed	Theoretical	Observed
MXF	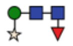	3810.6	3810.7	-	-	2478.9	2478.1
M2F	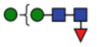	-	-	-	-	2508.9	2508.1
M2XF	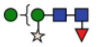	3972.6	3971.7	-	-	2640.9	2640.1
M3X	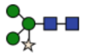	-	-	3871.3	3871.5	2655.9	2656.1
M3F	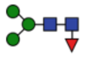	-	-	3886.3	3885.5	2670.9	2671.1
M3XF	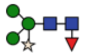	4134.6	4133.8	4018.3	4017.5	2802.9	2803.1
GNM2XF	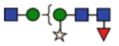	-	-	-	-	2843.9	2844.2
GNM3XF	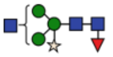	4337.6	4336.9	4221.3	4220.6	3005.9	3006.2
GAGNM3XF	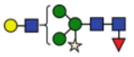	-	-	4383.3	4382.6	3167.9	3168.3
GN2M3XF	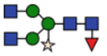	4540.6	4539.9	4424.3	4423.7	3208.9	3209.2
GAFGNM3XF	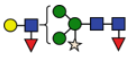	-	-	-	-	3314.9	3314.3
GAGN2M3XF	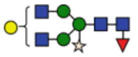	-	-	4586.3	4585.7	3370.9	3371.3
GAFGN2M3XF	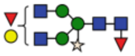	-	-	4733.3	4732.7	3517.9	3517.4
GA2GN2M3XF	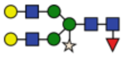	-	-	-	-	3532.9	3533.4
GA2FGN2M3XF	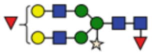	-	-	-	-	3679.9	3679.4
GA2F2GN2M3XF	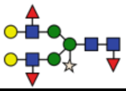	-	-	-	-	3826.9	3825.4

Supplementary Figure 2. Quantification of *N*-glycan structures on the purified GCase produced from wild type agroinfiltrated *N. benthamiana* leaves (MS mode)

Abbreviation	Structure	Mass-to-charge ratio (*m/z*)					
		N59		N146		N270	
		Theoretical	Observed	Theoretical	Observed	Theoretical	Observed
Core+GN	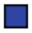	3166.6	3166.5	3050.3	3050.2	1834.9	1834.8
GN2	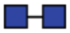	3369.6	3369.6	3253.3	3253.3	2037.9	2037.9
MGN2	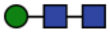	3531.6	3531.6	3415.3	3415.3	2199.9	2199.9
M2GN2	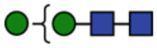	3693.6	3693.7	3577.3	3577.4	2361.9	2362.0
M3GN2	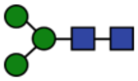	3855.6	3855.7	3739.3	3739.4	2523.9	2524.0
M4GN2	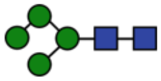	4017.6	4017.8	3901.3	3901.5	2685.9	2686.1
M5GN2	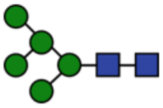	4179.6	4179.8	4063.3	4063.5	2847.9	2848.1

Supplementary Figure 3. Quantification of *N*-glycan structures on the purified GCase produced from ΔgntI agroinfiltrated *N. benthamiana* leaves (MS/MS mode)

Abbreviation	Structure	Mass-to-charge ratio (*m/z*)					
		N59		N146		N270	
		Theoretical	Observed	Theoretical	Observed	Theoretical	Observed
M2	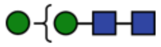	-	-	-	-	2361.9	2362.0
M3	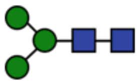	3855.6	3855.7	3739.3	3739.4	2523.9	2524.0
M4	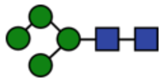	4017.6	4017.8	3901.3	3901.5	2685.9	2686.1
M5	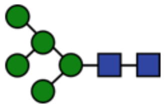	4179.6	4179.8	4063.3	4063.5	2847.9	2848.1

Supplementary Figure 4. Quantification of *N*-glycan structures on the purified GCase produced from ΔgntI agroinfiltrated *N. benthamiana* leaves (MS mode)
